# Using human-severe combined immunodeficiency (hu-SCID) mice as a model for testing allergenicity of genetically modified organisms (GMOs)

**DOI:** 10.1186/2045-7022-3-S3-P80

**Published:** 2013-07-25

**Authors:** R Lee, D Reiner, A Maczó, N Bakos, É Gelencsér, TJV Higgins, MM Epstein

**Affiliations:** 1 Division of Immunology, Allergy and Infectious Diseases, Experimental Allergy, Department of Dermatology, Medical University, Vienna, Austria; 2 Unit of Biology, Central Food Research Institute, Budapest, Hungary; 3 Plant Industry, Commonwealth Scientific and Industrial Research Organization, Canberra, Australia

## Background

GM peas expressing the alpha-amylase inhibitor (AAI) protein derived from common *Tendergreen* bean were created to protect them against infestation by pea weevils. Although animal models are used for studying allergenicity of GM containing foods, the correlation with human allergy remains controversial.

## Methods

Our aim in this study was to establish a humanized mouse model to study GMOs by adoptively transferring peripheral blood mononuclear cells from AAI-specific IgE-positive legume allergic and non-allergic individuals into SCID mice. These mice were then gavaged twice weekly for 1 month with GM pea, non-transgenic pea and Tendergreen bean seed meal suspensions or PBS. Subsequently, mice were administered 1 dose of the purified AAI protein or PBS intranasally as a read out for an *in vivo* allergic response. Seventy-two hours later, no eosinophilia or mucus hypersecretion were observed in the lungs of mice treated with PBS or fed the non-transgenic peas.

## Results

In contrast, hu-SCID mice derived from allergic and non-allergic donors had intense allergic lung inflammation and mucus over production illustrating that AAI-containing seed meal feeding primed for allergic immune responses. However, there were no differences in the responses between hu-SCID mice derived from allergic and non-allergic donors. Because both donors had anti-AAI antibodies, we hypothesize that they were both sensitized to AAI and thus indistinguishable when their cells were exposed to AAI during feeding in the humanized mice.

## Conclusion

This model provides new opportunities to investigate factors underlying the propensity to develop food allergy in individuals with antibodies to food allergens.

## Disclosure of interest

None declared.

